# VitalDB, a high-fidelity multi-parameter vital signs database in surgical patients

**DOI:** 10.1038/s41597-022-01411-5

**Published:** 2022-06-08

**Authors:** Hyung-Chul Lee, Yoonsang Park, Soo Bin Yoon, Seong Mi Yang, Dongnyeok Park, Chul-Woo Jung

**Affiliations:** Department of Anesthesiology and Pain Medicine, Seoul National University College of Medicine, Seoul National University Hospital, Seoul, Republic of Korea

**Keywords:** Medical research, Machine learning

## Abstract

In modern anesthesia, multiple medical devices are used simultaneously to comprehensively monitor real-time vital signs to optimize patient care and improve surgical outcomes. However, interpreting the dynamic changes of time-series biosignals and their correlations is a difficult task even for experienced anesthesiologists. Recent advanced machine learning technologies have shown promising results in biosignal analysis, however, research and development in this area is relatively slow due to the lack of biosignal datasets for machine learning. The VitalDB (Vital Signs DataBase) is an open dataset created specifically to facilitate machine learning studies related to monitoring vital signs in surgical patients. This dataset contains high-resolution multi-parameter data from 6,388 cases, including 486,451 waveform and numeric data tracks of 196 intraoperative monitoring parameters, 73 perioperative clinical parameters, and 34 time-series laboratory result parameters. All data is stored in the public cloud after anonymization. The dataset can be freely accessed and analysed using application programming interfaces and Python library. The VitalDB public dataset is expected to be a valuable resource for biosignal research and development.

## Abstract (summary)

**Table:** 

Measurement(s)	vital signs of patients during surgery • perioperative patient information
Technology Type(s)	Vital Signs Measurement • Electronic Medical Record
Factor Type(s)	vital signs data including various numeric and waveform data acquired from multiple patient monitors • perioperative patient information acquired from the electronic medical record system
Sample Characteristic - Organism	Homo sapiens
Sample Characteristic - Environment	hospital
Sample Characteristic - Location	South Korea

## Background & Summary

Intraoperative vital signs such as electrocardiography, blood pressure, percutaneous oxygen saturation, and body temperature are objective measures of physiologic function and are tracked with high-acuity patient monitors during surgery and anesthesia. These vital signs are usually used as-is, but sometimes converted into clinically useful secondary parameters developed through mathematical, engineering, and medical algorithms. Modern anesthesia widely adopts advanced patient monitors that present a variety of secondary parameters such as electroencephalogram-based anesthesia depth index, arterial pressure-derived cardiac output, and electrocardiography and photoplethysmography-based analgesia index. Numerous studies have shown that these secondary parameters are useful for optimizing patient care during surgery and greatly improve postoperative outcomes^1–3^.

Recent advances in machine learning technologies such as one-dimensional convolutional neural network allowed more accurate interpretation of the complex time-series biosignals^4^. The relationship between various vital signs was also elucidated using artificial intelligence resulting in practical high-performance algorithms in the medical field^5,6^. However, the lack of large-scale, high-resolution biosignal data required for machine learning has been a major obstacle to the development or improvement of biosignal algorithms. Electronic medical records (EMR) systems and automated anesthesia records (AAR) are important sources of biosignal big datasets, however, they have limited capabilities because (1) most EMR systems and AARs only store low time resolution data that are insufficient for interpretation of dynamic physiological changes during surgery; (2) essential waveform data such as electrocardiography, photoplethysmography, electroencephalography, and airway pressure waves are not stored on most systems due to cost or technical limitations, and (3) current recording systems do not fully support integrated recording of data from multiple devices^7,8^. In general, obtaining high-quality vital signs data in surgical patients is considered technically difficult or very expensive.

Previously, we developed the Vital Recorder program, a data capture software that records time-synchronized high-resolution data from various anesthesia devices including patient monitors, anesthesia machines, brain monitors, cardiac monitors, target-controlled infusion pumps, and rapid infusion system^9^. All parameters of multiple monitoring devices applied simultaneously to one patient are recorded as time-synchronized data tracks and stored as a single case file. Automatic recording function of this program has enabled massive collection of intraoperative biosignals in our tertiary, university hospital. The Vital signs DataBase (VitalDB) was constructed using (1) de-identified case files that were automatically recorded by the Vital Recorder program during daily surgery and anesthesia, and (2) perioperative patient information retrieved from our EMR system.

Unlike the previously reported public multi-parameter biosignal datasets^10–12^, the VitalDB is the first public biosignal dataset specifically focused on perioperative patient care and is characterized by containing multi-parameter high-resolution waveform and numeric data^13^. Since the VitalDB dataset was first released in 2017, it has been used for various big data research such as: deep learning for arterial pressure waveform-based cardiac output algorithm, deep learning-based pharmacokinetic-pharmacodynamic study of intravenous anesthetics, machine learning for bispectral index algorithm, statistical analysis of the relationship between intraoperative bispectral index and postoperative mortality, and deep learning algorithm to predict intraoperative hypotension from arterial waveforms^14–18^.

Perioperative clinical information, laboratory results and surgical outcomes in this dataset may facilitate a variety of clinical outcomes or clinical decision support studies. Studies that elucidate the relationship between biosignal parameters and clinical variables will also be feasible. For instance, the effects of intraoperative variables such as hypotension, hypothermia, and low cardiac output on clinical outcomes such as acute kidney injury, the length of hospital stay, or in-hospital mortality can be examined. The physiologic effects of various interventions such as vasoactive drugs, fluids, anesthetics, and anesthesia machine settings may be sought from the dataset. This dataset may simply be used as data samples for developing signal processing algorithms. However, we argue that this big data is better suited for a training dataset for machine learning of biosignals or for external validation of biosignal algorithms created using other datasets.

A final point to mention is the limitation that our data are from a single institution and a single race (Asian). Researchers should be careful as this can lead to overfitting of algorithms. As multicenter data can be a solution to this problem, we have released the Vital Recorder program and the VitalDB dataset for free. We hope that multicenter biosignal research for the development of general algorithms will be widely implemented in the future.

## Methods

The database includes vital signs data and related clinical information that were prospectively recorded during surgery. The patient information was retrospectively obtained from our hospital’s EMR system after surgery.

### Approval for data collection

The acquisition and free disclosure of the data was approved by the Institutional Review Board of Seoul National University Hospital (H-1408-101-605). The study was also registered at clinicaltrials.gov (NCT02914444). Written informed consent was waived due to anonymity of the data. Data collection was performed in accordance with relevant guidelines and regulations of the institutional Ethics Committee.

### Study population

Data were obtained from non-cardiac (general, thoracic, urological, and gynecological) surgery patients who underwent routine or emergency operation at Seoul National University Hospital, Seoul, Korea from Aug 2016 to Jun 2017. Of the 7,051 eligible cases, cases with local anesthesia (239), incomplete recording (279), and loss of essential data tracks (145) were excluded. Finally, 6,388 cases (91%) who received general anesthesia, spinal anesthesia, and sedation/analgesia were included in the dataset (Table 1).

**Table 1:** Dataset characteristics.

	General surgery (n = 4,930)	Thoracic surgery (n = 1,111)	Urology (n = 117)	Gynecology (n = 230)	Total (n = 6,388)
Demographic					
Sex (male)	2,524 (51%)	618 (56%)	101 (86%)	0 (0%)	3,243 (51%)
Age (years)	59 (48–68)	61 (52–70)	64 (58–72)	45 (35–55)	59 (48–68)
Height (cm)	162 (156–169)	163 (156–169)	168 (161–173)	159 (156–163)	162 (156–169)
Weight (kg)	60 (53–69)	61 (54–69)	69 (62–77)	59 (53–66)	61 (53–69)
Surgical approach					
Open	3,104 (63%)	190 (17%)	6 (5%)	65 (28%)	3,365 (53%)
Videoscopic	1,691 (34%)	889 (80%)	34 (29%)	140 (61%)	2,754 (43%)
Robotic	135 (3%)	32 (3%)	77 (66%)	25 (11%)	269 (4%)
Type of anesthesia					
General	4,650 (94%)	1,093 (98%)	117 (100%)	203 (88%)	6,063 (95%)
Spinal	246 (5%)	0 (0%)	0 (0%)	27 (12%)	273 (4%)
Sedation/analgesia	55 (1%)	18 (2%)	0 (0%)	0 (0%)	73 (1%)
Duration (min)					
Anesthesia	150 (90–245)	170 (110–220)	190 (80–230)	135 (95–185)	150 (90–240)
Surgery	110 (60–200)	115 (70–160)	145 (50–190)	95 (60–140)	110 (60–190)
Data	159 (100–259)	178 (124–236)	200 (91–238)	147 (105–198)	165 (103–251)
Anesthetic					
Sevoflurane	2,076 (42%)	255 (23%)	34 (29%)	172 (75%)	2,537 (40%)
Desflurane	1,012 (21%)	226 (20%)	106 (91%)	69 (30%)	1,413 (22%)
Propofol TCI	2,490 (51%)	996 (90%)	7 (6%)	5 (2%)	3,498 (55%)
Remifentanil TCI	3,639 (74%)	1,000 (90%)	86 (74%)	110 (48%)	4,835 (76%)
Device use					
TramRac-4A (SNUADC)	4,908 (100%)	1,104 (99%)	117 (99%)	226 (98%)	6,355 (99%)
Solar 8000 M	4,930 (100%)	1,111 (100%)	117 (100%)	230 (100%)	6,388 (100%)
Primus	4,915 (100%)	1,104 (99%)	117 (100%)	226 (98%)	6,362 (100%)
BIS Vista	4,282 (87%)	1,004 (90%)	84 (72%)	196 (85%)	5,566 (87%)
Orchestra	3,721 (76%)	1,005 (91%)	86 (74%)	115 (50%)	4,927 (77%)
Vigileo	85 (2%)	227 (20%)	32 (27%)	4 (2%)	348 (5%)
EV1000	598 (12%)	1 (0%)	0 (0%)	0 (0%)	599 (9%)
Vigilance II	63 (1%)	1 (0%)	0 (0%)	0 (0%)	64 (1%)
CardioQ-ODM+	29 (0.6%)	0 (0%)	0 (0%)	0 (0%)	29 (0%)
INVOS	33 (1%)	0 (0%)	0 (0%)	0 (0%)	33 (1%)
FMS2000	15 (0%)	0 (0%)	0 (0%)	0 (0%)	15 (0%)

Data is expressed as absolute numbers (%) or median (interquartile range).

Abbreviation: TCI = Target-controlled infusion.

### Dataset development

**Fig. 1:**
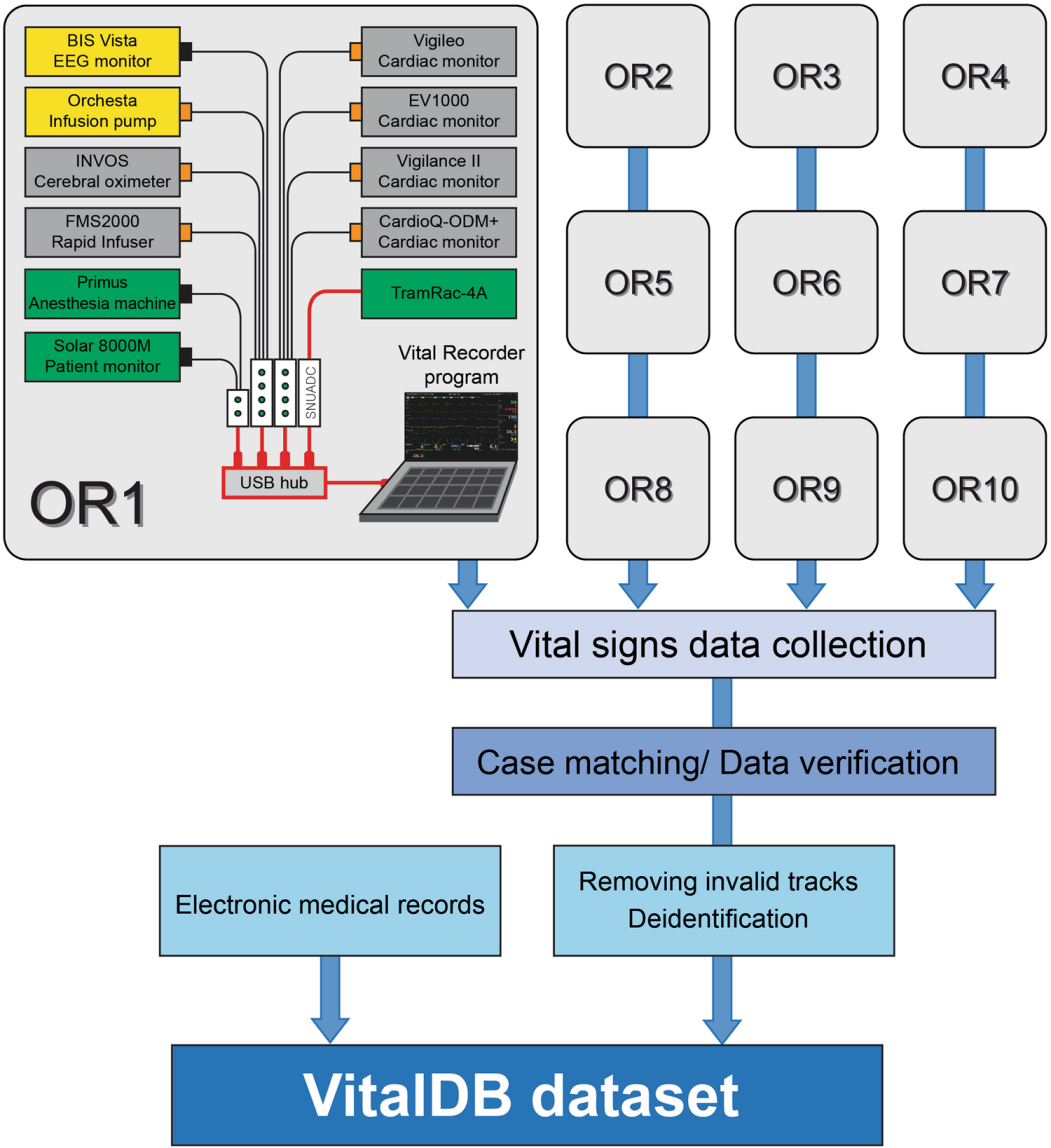
Schematic representation of vital signs data collection and VitalDB dataset creation. Vital signs data from multiple anesthesia devices are automatically recorded during surgery using the Vital Recorder program. The recorded files are checked for data adequacy and then combined with patient information from electronic medical records to create the VitalDB dataset.

These methods are expanded versions of descriptions in our related work^9^. All case files in this dataset were recorded using the Vital Recorder program (v 1.7.4). The laptop computer executing the Vital Recorder program was connected to multiple patient monitoring devices via serial cables (Fig. 1). Monitoring data from multiple anesthesia devices applied to one patient were recorded in one case file in a time-synchronized manner.

The same recording systems were installed in 10 out of 31 operating rooms to collect data over a year. The recording system operated for 24 hours every day, and case files of individual patients were automatically recorded separately. The case-by-case automatic recording was performed with the following method:

- When both heart rate and percutaneous oxygen saturation signals are detected, patient monitoring is considered to have started and case recording begins immediately.
- If the input of heart rate and percutaneous oxygen saturation signals disappears for more than 10 minutes according to the end of patient monitoring, the recording is automatically stopped.
  The data collection process was remotely monitored every day in real-time through web monitoring, and the integrity of the case-matched vital files was reviewed on a weekly basis. After verification of case-matched vital files (detailed in the Technical Validation section), track processing was performed using code for verified vital files.
- Tracks with all 0 values or less than 10 data samples were deleted.
- Waveform tracks without corresponding numeric tracks were deleted.
- Track name changes have been made for improving the usability of the dataset.
  - If a femoral arterial catheter was confirmed on the anesthesia records, the related arterial waveform and numeric tracks were renamed to from ART, ART_SBP, ART_DBP, and ART_MBP to FEM, FEM_SBP, FEM_DBP, and FEM_MBP, respectively.
  - “PUMP” in the PUMP_RATE and PUMP_VOL tracks has been changed to specific drug names, obtained from infusion pump data or anesthesia records (eg. EPI_RATE, PPF20_VOL).
  The demographic, surgical, anesthetic, preoperative, intraoperative and outcomes data of the patients were obtained from the EMR system and included in the dataset. The laboratory test results within 90 days before and after the anesthesia start time were extracted from the EMR, and all non-numeric characters were removed from the results. This information is organized in separate csv files in the dataset.
  Finally, de-identification of the dataset was performed before the release of the dataset.
- Instead of the actual patient number, random surgery case identifiers (caseid) were assigned to the cases (1–6,388); Individual identifiers of the hospital ID (subjectid) was also added for reoperation case identification (1–6,090).
- Since case-matched-and-renamed vital files no longer contain any patient identification information, only de-identification of the recording time was performed.
  - The surgery start and end times, and the anesthesia start and end times were extracted from the EMR and integrated to the event track of vital files.
  - The starting time point of the recording was set to “0” and the other time were converted to the relative time to the start point.

## Data Records

The dataset consists of intraoperative vital signs data (6,388 vital files in *vital* format), perioperative clinical information (clinical information.csv) and the laboratory results (lab results.csv) of 6,388 surgery cases. All data is accessible from an open data repository (VitalDB Open Dataset in *Open Science Framework*)^19^.

In brief, the dataset has the following characteristics:

- The dataset consists of intraoperative vital signs data and perioperative clinical information of 6,388 cases.
- Vital signs data includes up to 12 waveform and 184 numeric data tracks acquired from multiple anesthesia devices applied to patients during surgery. The total number of data tracks is 486,451 (average 87, range 16–129).
- Vital signs data have various time intervals according to the anesthesia devices, with a time resolution of 1–7 seconds for numeric data and 62.5–500 Hz for waveform data (Table 2). Each case file contains an average of 2.8 million data points.

**Table 2:** Devices and parameters in the data tracks.

Device	Device type	Company	Parameter	Data type	Number of parameters	Acquisition interval (sec)
TramRac-4A*	Patient monitor	GE healthcare	ECG, capnography, plethysmogram, blood pressures	Wave	6	1/500
Solar 8000 M	Patient monitor	GE healthcare	Heart rate, blood pressures, oxygen saturation, temperature, gas concentrations, etc.	Numeric	44	2
Primus	Anesthesia machine	Drager	Gas concentrations, volumes and flows, airway pressures	Wave; Numeric	2; 35	1/62.5 for waves; 7 for numeric values
BIS Vista (BIS 4.1 engine)	EEG monitor	Covidien	EEG waves, Bispectral index and related parameters	Wave; Numeric	2; 6	1/128 for EEG wave; 1 for numeric values
Orchestra	Target-controlled infusion pump	Fresenius Kabi	Target, plasma and effect-site concentrations; infused volume and infusion rate of drugs	Numeric	51	1
Vigileo	Cardiac output monitors	Edwards Lifesciences	Stroke volume and derived parameters	Numeric	5	2
EV1000	Cardiac output monitors	Edwards Lifesciences	Stroke volume and derived parameters		9	2
Vigilance II	Cardiac output monitors	Edwards Lifesciences	Cardiac output and derived parameters, temperature, oxygen saturation,		14	2
CardioQ-ODM+	Cardiac output monitors	Deltex	Stroke volume, cardiac output and related parameters	Wave; Numeric	2; 11	1/180 for waves; 1 for numeric values
INVOS	Cerebral/somatic oximeter	Covidien	Cerebral oxygen saturation	Numeric	2	5
FMS2000	Rapid infusion system	Belmont Instrument	Infused volume, infusion rate, temperatures, pressure	Numeric	7	every 2.875 mL infused

*Waveform data from TramRac-4A was recorded using an analog-to-digital converter (SNUADC) developed by authors.
- Data is not pre-processed because the real-world noise in the vital signs data is very essential to the development of practical monitoring algorithms.
- A total of 74 perioperative clinical information parameters and 34 time-series perioperative laboratory results are provided to help interpret the relationship with the intraoperative vital signs.

Since different anesthesia equipment was used for each patient, the data tracks are configured differently for each case file. Specifically, data from the patient monitor (Solar^TM^ 8000 M, GE healthcare, Wauwatosa, WI, USA) was taken from all patients, and analog signal (TramRac-4A, GE healthcare, Wauwatosa, WI, USA) data was acquired from most patients. Data from the anesthesia machine (Primus, Dräger, Lübeck, Germany) were recorded in most patients except for regional anesthesia cases. Data from the brain monitor (BIS Vista^TM^, Medtronic, Dublin, Ireland) were recorded in most patients undergoing general anesthesia and sedation/analgesia. Data from the target-controlled infusion pumps (Orchestra® Base Primea with module DPS, Fresenius Kabi AG, Bad Homburg, Germany) were recorded in all patients undergoing intravenous anesthesia and balanced anesthesia. The infusion pump data also includes infusion histories of various intravenous drugs. Cardiac monitors (Vigileo/FloTrac, EV1000 and Vigilance II monitors, Edwards Lifesciences, Irvine, CA, USA; CardioQ-ODM+, Deltex Medical, Chichester, UK), a rapid infusion device (FMS2000, Belmont instrument corporation, Billerica, MA, USA), and a cerebral/somatic oximeter (INVOS^TM^, Medtronic, Dublin, Ireland) were used at the anesthesiologist’s discretion. In conclusion, among the 196 parameters, 16–129 parameters were recorded for each case.

The clinical information file provides patient-related perioperative data to help interpret biosignal data (Table 3). This file consists of caseid and subjected, and 72 clinical parameters including case file information, demographic data, outcomes, preoperative laboratory data, and surgery and anesthesia related data. Among the parameters, “casestart” is the time the patient’s case file recording started, and the value is always “0”. All time-series data in the VitalDB dataset is anonymized in seconds using the casestart time as a reference point. Since the anesthesia start time (anestart) and anesthesia end time (aneend) are the times recorded at 5-minute intervals in the EMR, there may be a difference of several minutes from the start time (casestart) and end time (caseend) of the actual case recording.

**Table 3:** Parameters of clinical information.

Category (n)	Parameters	Description
ID (2)	caseid; subjectid	Randomly assigned case number (1–6,388); Deidentified hospital ID of patient
Case file information (6)	casestart; caseend; anestart; aneend; opstart; opend	Time of case recording start (0); Time of case recording end (s); Time of anesthesia start (s); Time of anesthesia end (s); Time of surgery start (s); Time of surgery end (s)
Outcomes (4)	adm; dis; icu_days; death_inhosp	Time of admission (s); Time of discharge (s); Postoperative length of stay in the intensive care unit (d); In-hospital mortality
Demographic (5)	age; sex; height; weight; bmi	Patient age (y); Gender; Height (cm); Weight (kg); Body mass index (kg/m^2^)
Surgery & Anesthesia (9)	asa; department; optype; dx; opname; approach; position; emop; ane_type	American Society of Anesthesiologists physical status classification (1–6); Surgical department; Surgical site or type; Diagnosis; Operation name; Surgical approach; Surgical position; Emergency surgery; Type of anesthesia
Preoperative morbidity (4)	preop_htn; preop_dm; preop_arrhythmia; preop_pft	History of hypertension; History of diabetes; Arrhythmia in the electrocardiogram; Result of pulmonary function test
Preoperative laboratory data (18)	preop_hb; preop_plt; preop_pt; preop_aptt; preop_na; preop_k; preop_gluc; preop_alb; preop_ast; preop_alt; preop_bun; preop_cr; preop_ph; preop_hco3; preop_be; preop_pao2; preop_paco2; preop_sao2	Hemoglobin level; Platelet level; Prothrombin time; Activated partial thromboplastin time; Sodium level; Potassium level; Glucose level; Albumin level; Asparate transferase level; Alanine transferase level; Blood urea nitrogen level; Creatinine level; pH; Bicarbonate level; Base excess; Partial pressure of oxygen; Partial pressure of carbon dioxide pressure; Arterial oxygen saturation
Intraoperative data (26)	cormack; airway; tubesize; dltubesize; lmasize; iv1; iv2; aline1; aline2; cline1; cline2; intraop_ebl; intraop_uo; intraop_rbc; intraop_ffp; intraop_crystalloid; intraop_colloid; intraop_ppf; intraop_mdz; intraop_ftn; intraop_rocu; intraop_vecu; intraop_eph; intraop_phe; intraop_epi; intraop_ca	Cormack’s grade; Oral or nasal airway; Endotracheal tube size; Double lumen tube size; Laryngeal mask airway size; Site of intravenous line 1, 2; Site of arterial line 1, 2; Site of central line 1, 2; Estimated blood loss; Urine output (mL); Amount of red blood cell transfusion (unit); Amount of fresh frozen plasma transfusion (unit); Infused volume of crystalloid (mL); Infused volume of colloid (mL); Propofol bolus dose (mg); Midazolam bolus dose (mg); Fentanyl bolus dose (mcg); Rocuronium dose (mg); Vecuronium dose (mg); Ephedrine dose (mg); Phenylephrine dose (mcg); Epinephrine dose (mcg); Calcium chloride dose (mg)

Finally, the laboratory results file contains 928,448 time-series data for 34 blood tests from 3 months before surgery to 3 months after surgery. Laboratory results are provided as a list of case identifier (caseid), blood test time (dt), test name (name), and value (result) for each test. Since the test time is a relative time expressed in seconds with the cases start time as a reference point, preoperative tests have negative time values.

Detailed descriptions and data availability of all vital signs tracks, clinical information, and laboratory results are uploaded in the open data repository (Suppl 1. VitalDB Parameters and Data Availavility.xlsx)^19^.

## Technical Validation

The case-matching and verification of the vital files was conducted as following:

- During recording, the connection status of anesthesia equipment was frequently assured by real-time remote monitoring.
- After surgery, the automatically recorded case files were retrospectively matched with the operation schedule retrieved from the EMR on a weekly basis.
  - Since the vital file name is automatically generated in the format of ‘operating room name_recording date_time (eg. OR1_170101_081005.vital)’, it is possible to specify the corresponding patient from the operation schedule.
  - Confirmation of matching was made by comparing the recording time in the vital file with the actual operation time of the patient in the EMR.
  The integrity of the vital files was validated as following:
- All case-matched files were separately loaded into the Vital Recorder program and visually verified by four anesthesiologists (authors YP, SBY, SMY and CWJ).
- If data tracks were found to be invalid during the data check, they were intentionally removed.
  - Inhalation anesthesia-related parameters during total intravenous anesthesia were deleted.
  - Waveform data tracks without corresponding numeric values have been removed.
- However, our dataset from real anesthesia partly contains the following signal noises. These noises have not been removed as they are essential elements for developing practical algorithms including data preprocessing.
  - Data loss of bispectral index, cerebral oximeter, electrocardiography, and plethysmography due to temporary sensor detachment.
  - Abnormal values of arterial pressures during blood sampling.
  - Electrocardiography and electroencephalography noises during electrocautery and electrophysiologic monitoring.

## Usage Notes

The use of the dataset for research and development begins with download of the data from the OSF repository (a total data volume of 103.4 GB)^19^. In this case, the research can be conducted using the python package.

### Python package (vitaldb)

The vital file is a binary file recorded with the Vital Recorder program and contain time-series records of vital signs. The specification of the vital file format is detailed a document in the open data repository (Suppl 2. Vital File Format.pdf)^19^.

A python package “vitaldb” that helps reading and writing of vital files is freely available on the Python Package Index.

There is a function named “load_case” to load track data from a single case file. The “load_case” function can be detailed as following:

- *Description:* Load multiple track data from single case.
- *Usage:* load_case (caseid, tnames, interval = 1)
- *Arguments*
  caseid: caseid to load.
  tnames: list or comma separated string of ‘device name/track name’.
  interval: time interval between data points. Default value is 1 second.
- *Usage Example*

load_case([‘SNUADC/ART’, ‘Solar8000/ART_SBP’], interval = 1/100)

load_case(‘SNUADC/ART,Solar8000/ART_SBP’, interval = 1/100)

There is a class called “VitalFile” in the vitaldb library that can help reading and writing vital files.

- *Description*: A class for reading and writing a vital file format.
- *Usage*: VitalFile (filepath, tnames)
- *Arguments*
  filepath: file path to read.
  tnames: list or comma separated string of ‘device name/track name’ to read
- *Usage Example*

vf = VitalFile(‘00001.vital’).to_vital(‘00001_copy.vital’)

vf = VitalFile(‘00001.vital’).to_numpy([‘SNUADC/ART’, ‘Solar8000/ART_SBP’], interval = 1/100)

vf = VitalFile(‘00001.vital’).to_pandas ([‘SNUADC/ART’, ‘Solar8000/ART_SBP’], interval = 1/100)

After reading the vital file with the “VitalFile” object, researchers can use “to_vital” method to save the data as vital file format again, or use “to_numpy” or “to_pandas” methods to get the samples of specific tracks as a numpy array or a pandas DataFrame.

### Web-based API

The use of Web-based API and cloud data may facilitate the research. Web-based API is provided for downloading track data and track lists from the endpoint URLs. Data can be accessed by entering the address into a web browser. All data track files are provided in csv format compressed with GZip (a total data volume of 113.2 GB).

- Clinical information file: https://api.vitaldb.net/cases
- Track list file: https://api.vitaldb.net/trks
- Data track files: https://api.vitaldb.net/*{tid}* where *tid* is a track identifier in the track list file.
- Laboratory results file: https://api.vitaldb.net/labs

Clinical information, Track list, and Laboratory Result files can be downloaded as csv format. In the track list file, each row consists of a case identifier (caseid), data track name (tname; Device name/Parameter name), and a 40-digit hexadecimal track identifier (tid) that is a unique address for an individual data track. Researchers can download and use the actual data track consisting of Time/Value by entering address information (tid) in the web-based API.

**Fig. 2:**
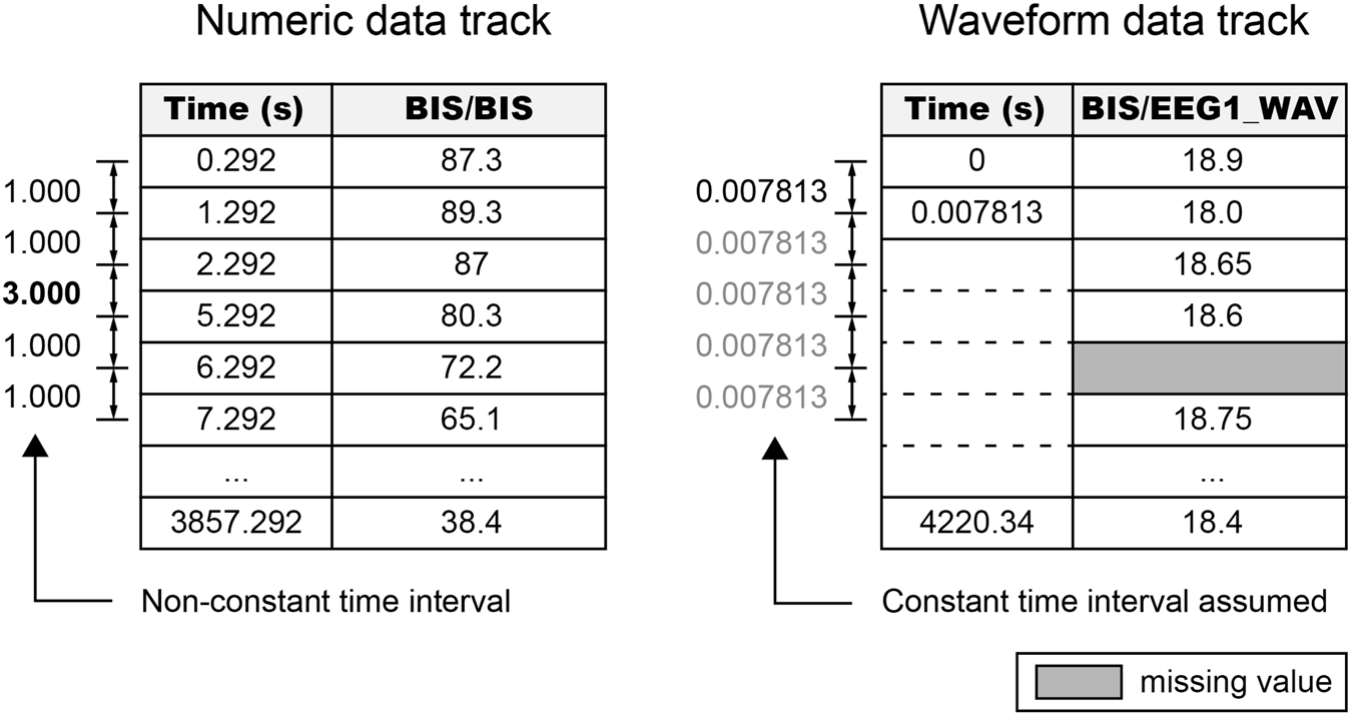
Formats of numeric and waveform data tracks (when using web-based API). The data track consists of two columns: Time and Value. In the numeric data track (left figure), the missing value rows are removed. In the waveform data track (right figure), rows with missing values are not removed but left blank. The time in the waveform data track is calculated from the start time and time interval using the monotonic increment function.

Data tracks are compressed csv files of the time-series data tracks extracted from the original vital files. The data track represents numeric or waveform data and consists of two columns: Time and Value (Fig. 2). The Time column contains the acquisition times of the measurement, and the Value column contains the measured values. In the numeric data track, the missing value rows have been removed, so the data collection time interval may be inconsistent. In the waveform data track, the time column has only three values: start time (0), time interval, and end time. The times of the waveform data track can be calculated in monotonic increments using the time interval value. Unlike the numeric data track, missing values are not removed but left blank in the waveform data track.

The general sequence of research using the Web-based API is as follows.

- Download the clinical information file and select caseids that satisfy the inclusion/exclusion criteria of research and development.
- Check the VitalDB API page for the names of the biosignal parameters (tname) needed for the research topic.
- Download the track list file, check the track identifiers (tids) of the data tracks that match the caseids and tnames, and download the actual track data using the API.
- Since the data tracks are time-synchronized with the casestart time (0) as the reference point, conduct research by converting multiple track data into an array on the same time axis.

## Data Availability

An open-source project facilitating the use of VitalDB dataset has been launched, and a lot of code written in C/C++, Python, Javascript, and R languages is currently available from Zenodo (10.5281/zenodo.6321507)^20^. Python codes that can be used as references of algorithm research are also available from Zenodo (10.5281/zenodo.6321522)^21^. The examples of sample codes for statistical analysis are as following: - General Characteristic: vitaldb_tableone.ipynb - Mortality: asa_mortality.ipynb - Acute Kidney Injury - mbp_aki.ipynb The examples of sample codes for artificial intelligence algorithms are as following: - Drug effect estimation using Long Short-Term Memory: ppf_bis.ipynb - Hypotension prediction using Long Short-Term Memory: hypotension.ipynb - Mortality prediction using Gradient Boosting Machine: predict_mortality.ipynb
